# Transforming early pharmaceutical assessment of genotoxicity: applying statistical learning to a high throughput, multi end point in vitro micronucleus assay

**DOI:** 10.1038/s41598-021-82115-5

**Published:** 2021-01-28

**Authors:** Amy Wilson, Piotr Grabowski, Joanne Elloway, Stephanie Ling, Jonathan Stott, Ann Doherty

**Affiliations:** 1 Functional and Mechanistic Safety, Clinical Pharmacology and Safety Sciences, R&D, AstraZeneca, Cambridge, UK; 2 Imaging and Data Analytics, Clinical Pharmacology and Safety Sciences, R&D, AstraZeneca, Cambridge, UK; 3 MAG-O, Manchester Airport, Manchester, UK

**Keywords:** Toxicology, Predictive markers, Computational science, Cell division, Chromosomes

## Abstract

To provide a comprehensive analysis of small molecule genotoxic potential we have developed and validated an automated, high-content, high throughput, image-based in vitro Micronucleus (IVM) assay. This assay simultaneously assesses micronuclei and multiple additional cellular markers associated with genotoxicity. Acoustic dosing (≤ 2 mg) of compound is followed by a 24-h treatment and a 24-h recovery period. Confocal images are captured [Cell Voyager CV7000 (Yokogawa, Japan)] and analysed using Columbus software (PerkinElmer). As standard the assay detects micronuclei (MN), cytotoxicity and cell-cycle profiles from Hoechst phenotypes. Mode of action information is primarily determined by kinetochore labelling in MN (aneugencity) and γH2AX foci analysis (a marker of DNA damage). Applying computational approaches and implementing machine learning models alongside Bayesian classifiers allows the identification of, with 95% accuracy, the aneugenic, clastogenic and negative compounds within the data set (Matthews correlation coefficient: 0.9), reducing analysis time by 80% whilst concurrently minimising human bias. Combining high throughput screening, multiparametric image analysis and machine learning approaches has provided the opportunity to revolutionise early Genetic Toxicology assessment within AstraZeneca. By multiplexing assay endpoints and minimising data generation and analysis time this assay enables complex genotoxicity safety assessments to be made sooner aiding the development of safer drug candidates.

## Introduction

Traditionally, toxicology assessments have been characterised by low throughput and high cost in vivo studies. However, there is now a drive to develop better predictive tools for toxicity studies, which would increase the accuracy of in vivo predictions and allow more informed assessments to be made, minimising unnecessary animal use and reducing the time and cost of pharmaceutical development. Advancements in screening approaches have led to rapid increases in the quantity and quality of data, which, when combined with the greater diversity of computational approaches to toxicology and risk assessment is driving the use of innovative approaches for the safety assessment of pharmaceuticals^1,2^.

Genetic Toxicology refers to the study of chemically or physically induced changes to DNA and chromosomes and the assessment of genotoxicity is a regulatory requirement during pharmaceutical development to evaluate potential carcinogenic risk. Genotoxicity can manifest in a variety of ways, including mutations (base substitutions for example), chromosomal aberrations and changes in chromosome number, all of which can induce carcinogenesis by increasing genomic instability^3^.

The complex nature and multiple mechanisms by which DNA damage can arise has led to the development of a battery of recommended assays for the assessment of pharmaceutical carcinogenic potential (ICHS2r1)^4^. The in vitro micronucleus (IVM) assay is one of the recommended cytogenetic tests for the assessment of chromosomal damage. The IVM assay assesses DNA damage at the chromosomal level by evaluating the presence of micronuclei. Micronuclei are small, membrane-bound nuclear bodies containing DNA, which are separated from the main nucleus during mitosis and can therefore be readily assayed using standard microscopy techniques and image analysis software^5–7^. Micronuclei can form from both chromosome fragments (clastogenic mechanisms), or whole chromosomes (aneugenic mechanisms).

Traditional IVM assays are low throughput and labour intensive (weeks/compound), require large amounts of compound (hundreds of milligrams) and do not offer mode of action information without the addition of supplementary endpoints (fluorescence in-situ hybridisation—FISH). Traditional IVM assays are therefore not suitable for compound screening early in pharmaceutical development when synthesis levels are low. To address some of the limitations outlined above, various approaches have been developed to simultaneously detect genotoxic responses whilst providing mechanistic information. These approaches include the integration of multiple in vitro assay endpoints^8^, Litron Laboratories’ MultiFlow multiplexed genotoxicity assessment method, which uses biomarkers and machine learning to classify genotoxic compounds^9^, and the ToxTracker system, a mouse stem cell-based reporter assay^10^. The combination of γ.H2AX and phosphorylated Histone H3 biomarkers for mode of action determination has also been championed in multiple assay formats, including flowcytometric and imaging based approaches and has shown promising result for mode of action determination in multiple studies^11^. To date however, no single approach addresses all of the limitations outlined above. The compound requirements for these assays are still relatively high (10′s-100′s mg) and some require sample processing to provide indirect measurements of DNA damage, i.e. via activation of a reporter genes in ToxTracker or γ.H2AX as an indication of double strand break repair without measuring micronucleus induction directly.

**Figure 1:**
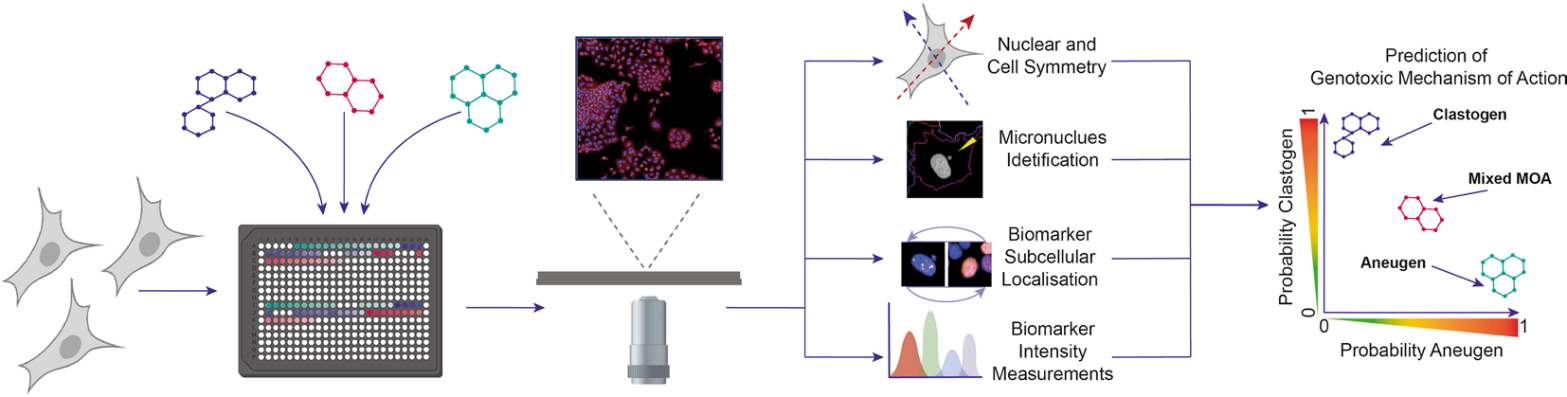
Representation of high throughput screening approach and methodologies to determine small molecule genotoxic potential. Illustrations in figure created with BioRender.com.

In this study we describe the development, optimisation and validation of a high-throughput screening assay which provides a comprehensive analysis of small molecule genotoxic potential (Fig. 1). By leveraging commercially available high-content imaging platforms, this assay concurrently detects micronuclei and other cellular markers associated with genotoxicity to provide mechanistic information. Combining these platforms with computational approaches and machine learning models has allowed the rapid and accurate assessment of the genotoxic potential of novel chemistry. By generating complex imaging data sets which can be probed for further information provides the potential to identify previously undetermined biomarkers of genotoxic risk. The assay provides the opportunity to screen multiple compounds simultaneously, for example early in pharmaceutical development when chemical design is not yet fixed and to predict for regulatory in vitro micronucleus assays that are required during pharmaceutical development*.*

## Results

### Assay development

Initial analysis identified the human lung epithelial cell line (A549) as an appropriate model system for this high-throughput assay; A549 cells are adherent, chromosomally stable (endogenous micronucleus frequency 3% ± 1.6% SD—data not shown) with a characteristic cell cycle profile. Other cell lines, including TK6, L5178Y and MCF10A were considered but dismissed based upon their adherence properties.

To align with the regulatory requirements for in vitro micronucleus assays an upper limit dose of 1 mM was selected^4,12^. The lower limit of 1 nM was selected following a literature review of doses at which genotoxic responses have been observed. Integrating Acoustic Droplet Ejection (ADE) technology with a 384 well plate format meant small compound amounts (~ 2 mg) were required to generate a 15-point half-log dose response (1 mM–1 nM), ensuring the suitability of the assay for the screening of compounds early in pharmaceutical development^13^ (S. Fig. S1). To minimise compound requirement dosing in 2% solvent was required, this did not induce significant morphologic or chromosomal changes in cells as illustrated by micronucleus analysis (S. Fig. S2a). A 24-h treatment with a 24-h recovery period was determined to be optimal for the determination of the genotoxic potential of both aneugens and clastogens (S. Fig. S2c,d).

The relationship between cytotoxicity and genotoxicity is complex, and since excessive cytotoxicity can manifest as DNA damage it is recommended that the maximal limit dose for analysis in cytogenetic assays is the dose at which approximately 50% cytotoxicity is observed^4,14^. In this assay the first dose at which > 50% reduction in cell number compared to the DMSO control was observed was the limit (cytotoxic concentration: CC50) dose.

### Assay validation

The assay integrates multiple endpoints to provide mode of action information. Hoechst, (nuclear DNA stain), is used to detect cell cycle changes, cell viability (cytotoxicity) as well as to measure micronucleus induction^15,16^. Phosphorylated Histone H2AX (γ.H2AX) was used to measure sites of DNA double strand break repair^17^, therefore, an induction of γ.H2AX foci indicated clastogenic damage. The presence of a kinetochore within a micronucleus indicates whole chromosome loss i.e. aneugencity, and was detected by antibodies targeted to kinetochore regions^18,19^. CellMask plasma membrane stain (ThermoFisher Scientific) was also used to define the cytoplasmic boundary.

**Figure 2:**
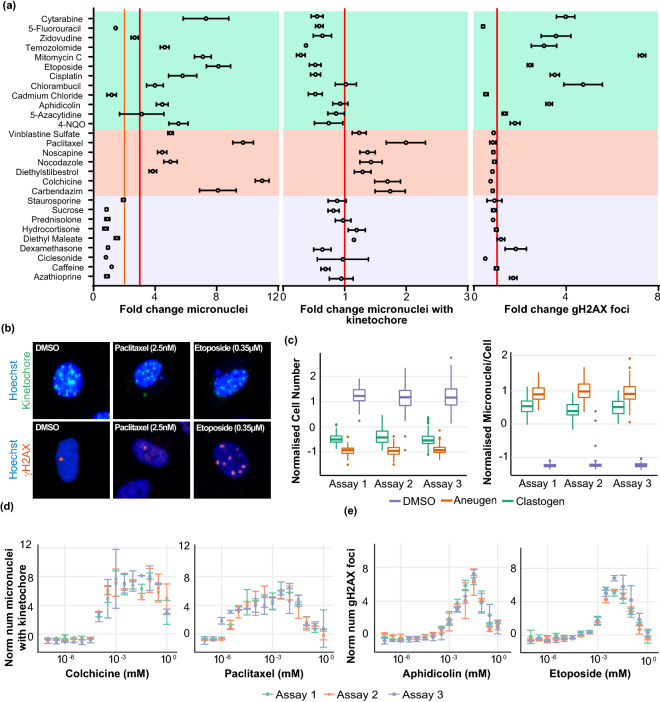
Validation of a high content in vitro micronucleus assay. (**a**) Normalised (c.f inter-plate DMSO wells) response (mean ± SEM) of validation compounds at the assay limit dose, illustrated are fold change in micronucleus per cell, fold change in kinetochore positive micronuclei per cell and fold change in γ.H2AX foci/nucleus. Non-genotoxic chemicals: purple shading, aneugenic chemicals: orange shading and clastogenic chemicals: green shading (n = 3, except Staurosporine and Zidovudine n = 2 and 5′Fluorouracil n = 4). (**b**) Representative images from plate control wells, cells treated with DMSO, Paclitaxel (2.5 nM) or Etoposide (0.35uM), DNA (Hoechst, blue), Kinetochore (CREST, green) and γ.H2AX foci (γ.H2AX, orange). (**c**) Intra-assay reproducibility for inter-plate controls and MOA assay endpoints. Variability in cell number and number of micronuclei (expressed as micronuclei/cell), when normalised to mean DMSO control across 3 independent assay runs (8 plates/run); negative control (DMS; purple-14 wells/plate), aneugenic control (Paclitaxel; green-12 wells/plate) and clastogenic control (Etoposide; orange-12 wells/plate). (**d**) Fold change (normalised to inter-plate DMSO controls) in kinetochore positive micronuclei following treatment with known aneugens: Colchicine and Paclitaxel from 3 independent assay runs. (**e**) Fold change (normalised to inter-plate DMSO controls) in γ.H2AX foci per nuclei following treatment with known clastogenic chemicals: Aphidicolin and Etoposide from 3 independent assay runs.

To validate the assay endpoints, the responses to a set of 28 validation compounds with well-defined genotoxic or non-genotoxic activities were examined in three replicate experiments (two technical replicates/experiment)^20,21^. Confocal images of cells treated with validation compounds were analysed for micronucleus induction using Columbus image analysis software. All genotoxic compounds, except 5′Flourouracil and Cadmium Chloride, induced significant dose-related increases in the number of micronucleate cells (Fig. 2a). For the known aneugens a concurrent increase in the proportion of micronuclei that contained a kinetochore was observed whereas a decrease was observed for all known clastogenic compounds (Fig. 2a,b). All known clastogenic compounds, except 5-Fluorouracil and Cadmium Chloride, induced significant increases in the number of γ.H2AX foci. As expected, no change in the number of γ.H2AX foci was observed upon treatment with known aneugens (Fig. 2a). The results for the primary assay endpoint, MN induction, correlated well with the results from previous in vitro studies, 86% sensitivity and 80% specificity.

When comparing assay runs, all-of the primary assay endpoints discussed above were highly reproducible. The induction of micronuclei in A549 cells upon exposure to Paclitaxel (2.5 nM) and Etoposide (0.35 µM), and the inter-plate control compounds, remained consistent (Fig. 2c), as did the dose-related induction of kinetochore positive micronuclei upon exposure to multiple different aneugenic compounds including colchicine and Paclitaxel (Fig. 2d). Exposure to clastogenic compounds (Aphidicolin and Etoposide) also induced remarkably reproducible dose related trends in γ.H2AX foci induction (Fig. 2e).

To establish thresholds for the assessment of the genotoxic activity of an unknown chemical entity, the observed magnitude of the responses of the validation compounds was compared to the inter-plate DMSO control wells. An unknown compound that induced greater than three-fold increase in micronucleus frequency was determined to give a positive genotoxic response in this assay; this threshold aligns with previously determined criteria for other high content micronucleus assays^6,22^ and all validation compounds were clearly positive using the criteria defined above, except the nucleoside analogue 5′Fluorouracil and the metal salt Cadmium Chloride. The antiviral agent zidovudine, induced a 2.7 (± 0.47) fold increase in micronucleus frequency in this assay, indicating a weak positive response. Multiple compounds that induced between a two and three-fold increase in micronuclei in this assay were tested in a regulatory in vitro micronucleus assay in L5178Y mouse lymphoma cells. All these compounds gave negative results according to the criteria set out in the guidelines, providing further support for the utilisation of a > 3-fold cut off for a positive response in this assay (S. Fig. S2b).

### Data analysis

**Figure 3:**
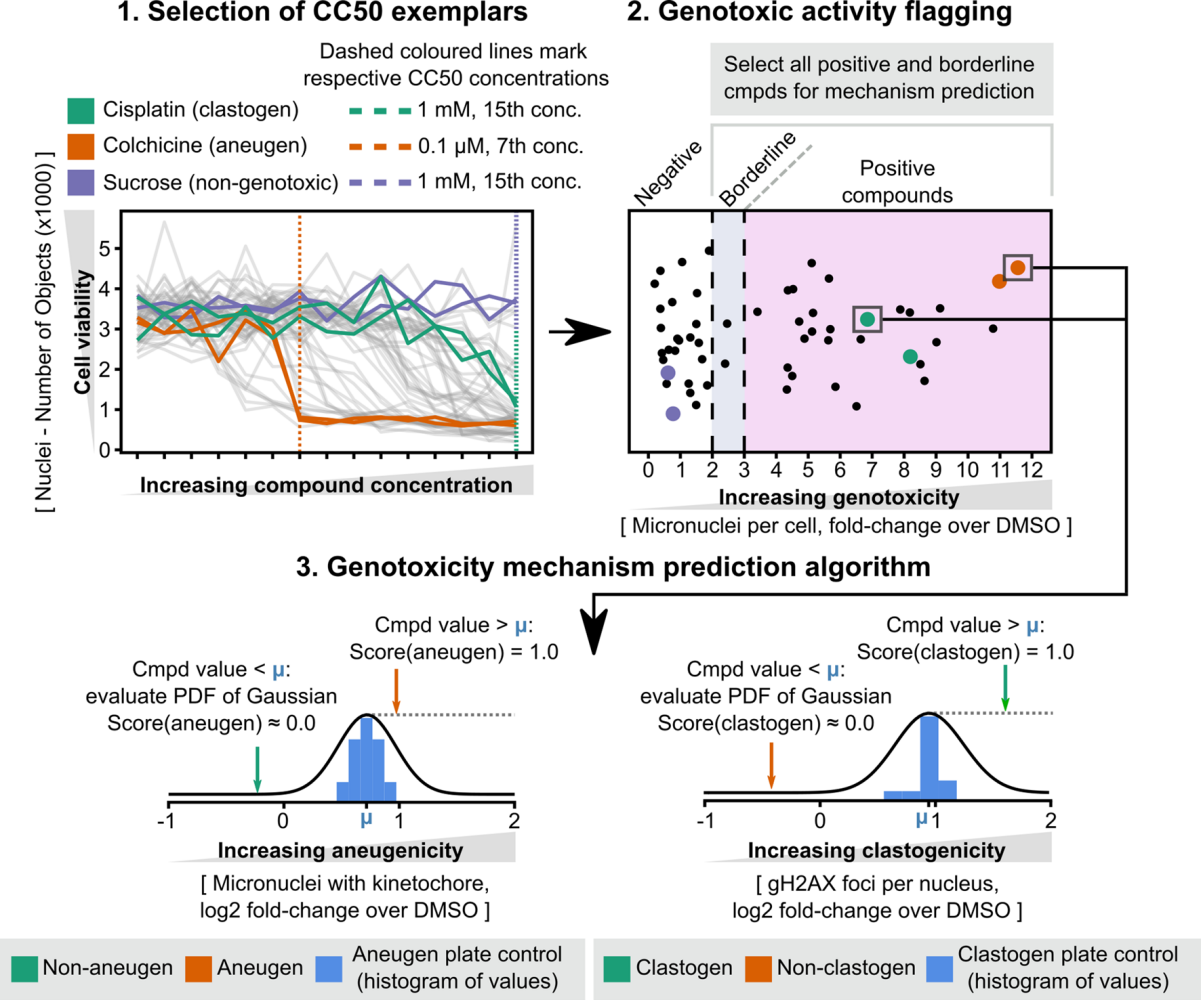
Statistical analysis workflow for automated prediction of genotoxicity for validation compounds. (1) Section of CC50 exemplars: the first concentration at which a greater than 50% reduction in cell number is observed or in the case of no cytotoxicity the highest concentration, is selected. Highlighted are Colchicine (Orange:0.1uM), Cisplatin (Green: 1 mM) and Sucrose (Purple: 1 mM). (2) Genotoxicity flagging: the frequency of micronuclei compared to average inter-pate DMSO control for each validation compound is shown, compounds with ≥ 2–< 3-fold increase in micronuclei are flagged as borderline and compounds > 3-fold increase in micronuclei classed as positive. Highlighted are replicate responses of Colchicine (Orange), Cisplatin (Green) and Sucrose (Purple). (3) Genotoxicity mechanism prediction. Gaussian distribution of inter-plate aneugen and clastogen control well responses for (**a**) the proportion of micronuclei with a kinetochore and (**b**) number of H2AX foci/nucleus respectively are plotted, responses for colchicine (orange: aneugen) and cisplatin (green: clastogen) are represented on these curves.

To further increase the throughput of the assay, automated data analysis methods were developed and employed to determine compound genotoxic potential without human data interpretation. The statistical analysis workflow consisted of three separate processes; initially the relevant compound concentrations to be used for subsequent analysis (CC50 exemplars) were determined. The data at these concentrations were then utilised to perform a genotoxicity flagging routine based solely on micronucleus frequency and finally mode of action was predicted for the genotoxic compounds (Fig. 3).

In the example shown here, the CC50 dose, or the first dose at which a greater than 50% reduction in cell number was observed for the pro-typical aneugen colchicine (0.1 µM) and for the clastogen Cisplatin (1 mM) are shown. Sucrose, which was utilised as a negative control in this study, did not exhibit any cytotoxicity therefore 1 mM was selected as the exemplar dose for further analysis.

The frequency of micronuclei (compared to inter-plate DMSO control wells) at the selected dose was then plotted, the positive and borderline compounds, as determined using the thresholds defined above (in this case; Cisplatin and Colchicine) were then selected for mechanism assessment.

To enable the assessment of compound mode of action Gaussian curves were fitted using the plate-specific aneugenic and clastogenic control compounds (Paclitaxel and Etoposide respectively) using the values of their hallmark feature for aneugenicity and clastogenicity (numbers of micronuclei with kinetochores and numbers of γ.H2AX foci per nucleus, respectively).

A simple rule-based model was applied to predict mode of action; if a compound value for the hallmark feature was larger than mean of the plate controls then the compound received a maximal score for being an aneugen or a clastogen. However, if the compound value was below the plate control mean for that feature, then the probability density function (PDF) was evaluated at that point. The resulting likelihood was then divided by the maximal likelihood of the respective Gaussian function, creating a “mechanism score” (Score) bounded between 0.0 and 1.0. Using these methods, Colchicine was given a maximal score of 1.0 of being an aneugen and Cisplatin a score of 1.0 of being a clastogen.

### Validation of additional assay endpoints

The use of image analysis software (in this case, Columbus Image Data Storage and Analysis system) allowed the multiparametric comparison of numerous phenotypic end points and the images were further analysed for additional endpoints to form complex imaging data sets.

Changes in cellular and nuclear morphology, including but not limited to; nuclear and micronuclear symmetry and roundness were examined for all fluorophores. Intensity and morphology measurements were recorded for each biomarker, for example, foci size, shape, intensity and symmetry for the γ.H2AX antibody.

**Figure 4:**
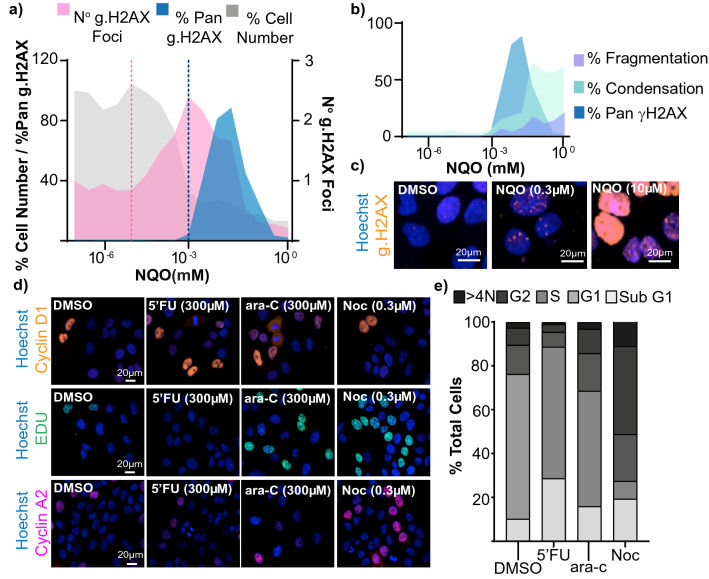
Validation of additional assay endpoints. Effect of A549 cell treatment with increasing concentrations of NQO on γ.H2AX phenotypes. (**a**) Quantification of the % of cells with pan γ.H2AX staining (blue), relative toxicity, illustrated by reductions in cell number (grey) and the average number of γ.H2AX foci per nucleus (pink). Horizontal lines illustrate the dose at which increases in γ.H2AX foci (pink) and % pan γ.H2AX stained cells were initially observed. (**b**) Quantification of the % of cells with pan γ.H2AX staining (blue), and nuclear condensation (green) and fragmentation (purple) phenotypes as indicators of cell death. (**c**) Representative immunofluorescence images of A549 cell nuclei treated with 0, 0.3 µM and 10 µM NQO (scale bar 20 µM), DNA (Hoechst, blue) and γ.H2AX (orange). Validation of use of Hoechst intensity for cell cycle analysis. (**d**) Representative immunofluorescence images of cells treated with DMSO (control), 5-Fluorouracil (5′-FU) (300 µM) Cytarabine (ara-c) (300 µM) and Nocodazole (0.3 µM); DNA (Hoechst, blue) with cyclin D1 (orange) EDU-GFP (green) or Cyclin A2 (pink). (**e**) Cell cycle analysis based on Hoechst intensity analysis of A549 cells treated for 48 h with DMSO (control), 5-Fluorouracil (5′-FU) (300 µM) Cytarabine (ara-c) (300 µM) and Nocodazole (Noc) (0.3 µM).

Unlike flow-cytometry-based methods, this assay can discriminate between γ.H2AX foci and pan-nuclear staining (Fig. 4a), providing an opportunity to assess the sub-nuclear kinetics of γ.H2AX accumulation after compound treatment. γ.H2AX foci are formed at sites of double strand breaks and it is widely accepted that the number of foci are directly proportional to the number of breaks, and so, as in this assay, can therefore act as a quantifiable index of DNA damage^23^. By comparison pan-nuclear staining has been observed upon the induction of intense replication stress and precedes irreversible cell death in this context^24^. More broadly pan-nuclear γ.H2AX staining has been associated with toxicity, cell death and apoptosis^25^. The ability of this imaging based method to discriminate between these phenotypes is illustrated upon treatment with 4-Nitroquinoline 1-oxide (4NQO^26^) which induced increases in γ.H2AX foci at lower concentrations than pan γ.H2AX stained nuclei, increases in which corresponded with the induction in markers of cell death (nuclear condensation and fragmentation) (Fig. 4b,c).

The analysis of cell cycle changes is an important consideration when evaluating genotoxic mode of action and can ascertain differential cytotoxic and cytostatic responses. The assay obtains Hoechst intensity measurements at the single cell level which enabled the generation of cell cycle profiles using a novel algorithm based upon those utilised for flow cytometry cell cycle analysis^27^. To validate this algorithm unsynchronised A549 cells were dosed with well characterised cell-cycle modulators for 48 h and the responses, as detected by the algorithm and were verified by immunofluorescence staining with cell-cycle specific markers, Cyclin D1 or Cyclin A2 (Fig. 4d).

As expected, treatment with 5-Fluorouracil (5′FU) (300 µM), an inhibitor of thymidyl synthase activity during S phase^22,28^ led to a decrease in the proportion of cells classified as S phase and a concurrent increase in cells in G1, as well as an increase in apoptotic cells, as indicated by the increase in the Sub G1 cellular fraction (Fig. 4e). Treatment with the nucleoside analogue Cytarabine (ara-c) (300 µM) for 48 h, induced a cell-cycle arrest at S phase. In line with its mode of action, treatment with Nocodazole (Noc) (0.3 µM), an inhibitor of microtubule-dependent cellular processes such as mitosis, lead to an increase in the proportion of cells in G2 (Fig. 4e).

Cyclin D1 levels increase during G2 phase, are maintained through mitosis and G1 phase, and decline when DNA synthesis begins^29^, whereas levels of Cyclin A2 increase through S Phase^30^ peak mid S phase and decline late in G2^31,32^. For all the cell-cycle changes mentioned above, concurrent changes in cell-cycle phase specific markers were observed. In addition, an increase in the percentage of cells with newly synthesised DNA, as indicated using a 5-Ethynyl-2´-deoxyuridine (EdU) incorporation assay, was also noted with nocodazole and Cytarabine (Fig. 4d), indicating the suitability of the automated cell cycle analysis method for utilisation in this assay.

## Discussion

By combining high-throughput screening technologies with multiparametric image analysis methods and computational approaches in a single assay we can reliably indicate the genotoxic potential and mechanism of action of an unknown compound. In the context of pharmaceutical development this assays allows the complex assessment of genotoxic potential to be made early in the drug development pipeline, when chemistry is not yet fixed, multiple compounds from different series are under evaluation, compound availability is limited and compound design can be influenced. The assay is currently used to predict for the regulatory standard in vitro micronucleus assay that is required for pharmaceutical development and therefore in turn this assay can be used to provide a mechanistic understanding of the genotoxic potential of a small molecule in vivo^33^*.* Small molecules assessed using this assay within AstraZeneca that are selected as candidate compounds are further assessed using the regulatory test battery, as shown here the results from this assay correlate well with the results observed in the regulatory assay and thereby this assay provides the opportunity to stratify compound selection. This assay may also be utilised outside of the pharmaceutical environment, the methodologies discussed here can be applied to the assessment of any unknown chemical entity and can be adapted for the assessment of, for example, agrochemicals. In addition, the endpoints discussed in this assay can also utilised more widely, for instance in oncology research, to assess the efficacy of potential treatments particularly those targeting the DNA Damage Response (DDR) pathways.

To determine the dosing schedule for this assay we investigated the responses of known aneugens and clastogens after different treatment and recovery regimens (24 or 48-h continuous treatment or a 24-h treatment + 24-h recovery. The inclusion of a recovery period has been shown to aid the detection of aneugenic compounds^34^. In this assay, all known genotoxic compounds induced significant increases in micronuclei with all treatment regimens, however, the magnitude of genotoxic response (micronucleus frequency) for the aneugenic compounds increased with the inclusion of a recovery period whilst the detection of clastogens was not adversely affected. Therefore, the 24-h treatment plus 24-h recovery regimen was selected.

The assay was validated with a set of well-defined genotoxicants (clastogens and aneugens) and non-genotoxicants. All, bar two (5-Fluorouracil and Cadmium Chloride) of the known genotoxicants tested were determined to be genotoxic according to the acceptability criteria for a positive in this assay i.e. a ≥ 3-fold induction of micronuclei at the limit dose for cytotoxicity^4,33^. The results from the assay correlated with published in vitro micronucleus data from other mammalian cell lines. Zidovudine, an antiviral agent induced a weak positive response in this assay, this is in agreement with previously published data for in vitro micronucleus assays in L5178Y mouse lymphoma cells, where a weak positive result was obtained only at the highest concentrations tested (4000–5000 μg/ml)^35^. The principal advantage of this assay is the simultaneous detection of DNA damage mode of action and resulting cell fate. Mode of action assessments of genotoxicity can be associated with carcinogenic risk^36^. Within the pharmaceutical industry acceptable thresholds can be established for compounds that are considered non-DNA reactive e.g. aneugens^37^. This is less accepted for a DNA reactive compounds^38^. However, the generation of high content quantitative dose response data, such as that in this assay, could enable safe dose margins or thresholds to be established for compounds using benchmark dose considerations^38^.

All genotoxic agents tested in this assay elicited the expected response except 5-Flurouracil and Cadmium Chloride. Cadmium Chloride is an inorganic carcinogen; the mechanisms that lead to cadmium induced carcinogenesis are complex and include aberrant gene expression, inhibition of DNA damage repair, induction of oxidative stress and apoptosis, these mechanisms have been shown to depend on dose and treatment duration^39^. Although non-genotoxic mechanisms such as the upregulation of intracellular signalling pathways leading to increased mitogenesis have been proposed to be a major contributor to the carcinogenic potential of Cadmium in vivo^40^, cadmium has been shown to induce micronuclei and DNA damage in vitro when solubilised in water^41–43^. In the development of this assay, all test compounds were solubilised in DMSO, although the solubility of cadmium chloride in DMSO (1800 g/l (25 °C) is similar to in water [1400 g/l (25 °C)], it is possible that the acoustic dosing technologies utilised in this assay may not be compatible with dosing metal salt solutions. In multiple cell lines, Cadmium induced DNA damage is observed at relatively high doses that are associated with cytotoxicity (500, 1000 and 2000 µM)^44^ furthermore A549 cells have been shown to exhibit tolerance towards cadmium induced cell death when compared to other lines (HEK293, HC116p53wt and HC116p53^−/−^ and CHO-9)^45,46^, highlighting the potential cell line and dose specific effects of this compound that may contribute to the lack of response observed in this assay. To assess this prolonged treatment regimens, with and without the inclusion of a recovery period, as well as a broader dose range could be assessed.

For all known clastogens tested, except for 5-Flurouracil and Cadmium Chloride, an increase in micronucleus frequency and in nuclear γ.H2AX foci was observed; there was also a marked decrease in proportion of kinetochore-containing micronuclei compared to the DMSO control, potentially highlighting this feature as a complementary indicator of clastogenicity. 5-Fluorouracil, is an analogue of uracil and is readily converted to a series of active metabolites in cells, which can be incorporated into RNA and DNA, disrupting synthesis as well as inhibiting the nucleotide synthetic enzyme thymidylate synthase, limiting the availability of thymidylate, which is necessary for DNA replication and repair^47^. The lack of observed response for 5-Fluorouracil may therefore reflect the mechanism by which 5-Fluorouracil exhibits genotoxic effects, γ.H2AX foci are not a direct measure of DNA damage rather of the DNA damage response associated with double stand break repair and therefore do not reflect all types of DNA damage e.g. single strand DNA breaks, replication fork stalling etc.^48^. The inclusion of a compound-free recovery period in this assay, during which double strand breaks may be repaired may also contribute especially considering γ.H2AX formation has been shown to be rapid, for example foci formation after ionizing radiation reaches a maximum after 30 min^14^. It cannot be ruled out however, that the difference in sensitivity observed in this assay compared to others reported in the literature^11^ may be due to a cell-line specific effect as demonstrated by Khuory et al. when comparing magnitude of γ.H2AX induction after treatment with 5-Fluorouracil in 4 cell lines^49^. Differences in DNA repair capacities or drug transporters in A549 cells compared to other commonly utilised cells lines may also contribute to this response. As increases in γ.H2AX foci are correlated with the transition in to mitosis (G2/M) in the cell cycle^17^, one must consider that increases in γ.H2AX foci may also indicate increased cell proliferation or cell cycle block. To further confirm the γ.H2AX foci increases observed in this assay are due to damage other markers for DNA damage response such a 53BP1 could also be assessed^25,50,51^.

For all the known aneugens tested, an increase in the proportion of kinetochore-containing micronuclei was observed and interestingly, a concurrent decrease in the number of nuclear γ.H2AX foci. This result corresponds with previously reported results from^52^ who measured γ.H2AX using whole cell ELISA and found that a decrease or no change in γ.H2AX was observed for aneugens at concentrations that induced micronuclei. Although the evaluation of the presence of a centromeric signal in micronuclei is accepted as a reliable method for assessing genotoxic mode of action, in other published genotoxicity screening assays, phosphorylated histone-H3, a biomarker of mitotic cells (aurora kinase family mediated phosphorylation of Serine 10 is required during mitosis for chromosome segregation and condensation^53^), has been utilised to determine an aneugenic mode of action^11,54^. In the assay presented here, a comprehensive analysis of the ability of a compound to induce micronuclei via an anagenic modes of action is generated by combining the image analysis process implemented in Columbus and the cell cycle profile analysis which allows the quantification of both the number of mitotic cells and number of micronuclei containing a kinetochore as well as compound induced cell cycle blocks.

By applying automated screening technologies^13^, this assay has significantly increased throughput compared to traditional in vitro micronucleus assays; we have generated data from hundreds of compounds in a short period of time (> 1000 compounds screened in 14 independent assay runs). The ability to test multiple compounds from various chemical series and compare the magnitude of micronucleus response (and other genotoxic endpoints) to on and off target efficacy provides the opportunity to determine chemical sub-structures that may be associated with increased carcinogenic risk. There is a long history of rule-based in silico models for genotoxicity prediction and there multiple commercially available QSAR and machine learning models for the prediction of mutagenic (Ames positive) compounds^55–57^, however, models for prediction of other mechanisms of DNA damage are very limited. Utilising the high content data generated from this assay may provide the basis for the development of such models.

An important consideration for the assessment of genotoxicity is the metabolic competence of a test system as some test substances require transformation to form a DNA reactive metabolite. This assay uses A549 cells, which have been shown to have limited metabolic capacity, specifically A549 cells have been shown to express both P450 IA1 and P450 IIB6, suggesting a capacity for phase I oxidative metabolism^58^, however the specific metabolic capacity of these cells, and their ability to perform phase II metabolism is not clear. To address this limitation, this assay could be supplemented by the addition of an exogenous metabolic activation system, such as the S9 fraction from the homogenized livers of chemically induced rats. S9 fraction has been shown to induce significant cytotoxicity in cell-based assays^59^ a phenomenon we have observed with A549 cells. Further approaches such as the use of a hepatoma cell lines and terminally differentiated HepaRG cells are also currently being explored.

Understanding the potential on and off target genetic toxicology effects of a small molecule is particularly important for classes of compounds where promiscuity is known to be an issue, and can influence the chemical design of, for example kinase inhibitors. Olaharski et al.^60^ compared kinase inhibition to micronucleus frequency and identified a panel of 21 kinases predictive of micronucleus induction. By combining the assay we have developed here with, for example CRISPR knock out cell lines and phenotypic siNRNA and CRISPR screening approaches, there is the potential to aid in the identification of further novel molecular targets associated with genotoxic risk^61^.

The variability between assay runs was very low; dose responses observed for individual chemicals were highly reproducible for all the assay endpoints, even those measured at the single pixel level (kinetochore). This reproducibility may, in part, be facilitated by the use of robotic automation, which can enable improvements in the consistency of assay timings and in the control of plate handling; however, although the assay discussed here is fully automated from dosing to fixation, manual handling of plates can also be employed to reduce instrumentation cost and therefore enable transfer of similar assays to facilities without automation.

The use of immunofluorescence and confocal image analysis provides several benefits over the current commercially available flow-cytometry-based multi end-point genotoxicity assessment assays. By utilising image analysis software and single cell intensity measurements of nuclear stains, well characterised methods from flow cytometry applications can be adapted and applied to determine cell cycle profiles from images. This method allows the analysis of cell cycle and cytostatic events in one channel without the use of further immunofluorescence markers and enables the direct assessment of cell cycle profiles alongside genotoxic and mode of action endpoints. The ability to directly measure multiple end-points, in situ, in real time and at the single cell level allows a more robust assessment of mode of action and the tracking of the impact of DNA damage throughout the cell cycle. This high-content assays’ ability to distinguish between apoptosis, and both γ.H2AX foci and pan γ.H2AX provides further information regarding the mode of action, as a sudden the change from foci to pan γ.H2AX can indicate replication catastrophe^62^. The high content images generated from this assay proved unprecedented levels of information and the potential to utilise these images to develop deep learning models, such as CNN (convolutional neural networks) to provide direct predictions of genotoxic potential without image analysis software is currently being explored.

By applying data analysis approaches and simple rule-based models we were able to determine compound genotoxic potential from image analysis data sets without manual data interpretation. The advantage of applying these techniques and utilising the respective Gaussian function approach discussed here is that the effect of assay-to-assay variability is minimised (by fitting curves to plate-specific controls), potential human bias is removed and the throughput of the assay is further increased. Moreover, the methods discussed here can be applied to data from various image analysis platforms and are not limited to the complex data sets generated in this screen. When compared to manual interpretation the model developed here classified with 95% accuracy, the aneugenic, clastogenic and negative compounds within the data set (Matthews correlation coefficient: 0.9), reducing analysis time by 80% whilst concurrently minimising human bias.

In conclusion, by combining high throughput screening, multiparametric image analysis and machine learning approaches we can generate complex genotoxicity safety assessments of early chemistry from a single assay, ensuring the development of safer drugs and transforming the assessment of genotoxicity within AstraZeneca.

## Materials and experimental proceedures

### Reagents

A549 Cells (American Type Culture Collection (ATCC), cat. No. CCL-185). All reagents and validation compounds were purchased from Sigma Aldrich unless otherwise stated.

### Validation compound selection

Validation compounds were selected from the recommended list of chemicals for the assessment of the performance of new or old improved genotoxicity assays^20^ and from other published assays developed for the assessment of genotoxicity^9,10,54^.

### Cell culture

A549 Cells were cultured in Roswell Park Memorial Institute (RPMI) 1640 media supplemented with 2 mM l-Glutamine (Gibco), 10% v/v Foetal Calf Serum (FCS) (Gibco) and penicillin (100 units)-streptomycin (0.1 mg/ml). Cells were maintained sub-confluent prior to treatment. All incubations prior to fixation were at 37 °C, 5% CO_2_ in a humidified incubator.

### Screening assay

A549 cells were seeded at 750 cells/well in 384-well CellCarrier-384 Ultra microplates (PerkinElmer) 24 h prior to treatment. An integrated robotic system from HighRes BioSolutions controlled by Cellario software (HighRes BioSolutions) was used to perform the assay. A Multidrop Combi (ThermoFisher Scientific) was used to dispense cells and antibody solutions. Aspiration and wash steps were completed using a BioTek EL406 plate washer (BioTek).

### Compound treatment

A Labcyte Echo 555 Acoustic Dispenser (Labcyte) was used to dose a 15-point half-log dose response (1 mM–1 nM, solvent 2% v/v) according to plate map design in S. Fig. S1. Inter-plate control compounds were as follows; [Paclitaxel (aneugen), 12 wells 2.5 nM) and Etoposide (clastogen), 12 wells (0.35 μM)]. Cells were incubated for 24 h prior to washing with media and a further 24-h incubation.

### Fixation and immunofluorescence

Cell were fixed in 4% paraformaldehyde (PFA) for 30 min at room temperature, prior to washing three times with phosphate buffered saline (PBS) followed by permeabilisation and incubation in blocking solution (PBS with 1.1% BSA and 0.1% Triton X-100) for one hour at room temperature. Cells were then incubated overnight at 4 °C with the following antibodies: anti-Centromere Antibodies (derived from human CREST patient serum (1:1000, Antibodies Incorporated)) and anti-γ.H2AX [(anti-phospho-Histone H2AX (Ser139), mouse) clone JBW301, 1: 10,000, Millipore] prepared in PBS block. Cells were washed three times with PBS and incubated at room temperature for one hour with secondary antibodies and DNA and cellular stains prepared in PBS block: [Goat anti-human Alexa 488 (1:500, ThermoFisher Scientific), Goat anti-mouse Alexa 555 (ThermoFisher), HCS CellMask Deep Red Plasma Membrane Stain (1:20,000, ThermoFisher Scientific) and Hoechst 33342 (1:1000, Invitrogen)]. Cells were washed with and then stored in PBS with ProClin 300 prior to imaging.

### Confocal microscopy

Cells were imaged on a confocal scanner (Cell Voyager 7000 (Yokogawa Inc.)) using 20× objective. Eight identical fields of view were captured per well in a single focal plane as determined by the integrated software focus algorithm in the Hoechst channel (455 nm). An ACell benchtop robot (HighRes BioSolutions) controlled by Cellario software (HighRes BioSolutions) was used for plate handling.

### Image analysis

Image analysis was completed using Columbus Image Data Storage and Analysis System (PerkinElmer), image analysis software, scripts and building blocks and each assay run was calibrated using positive and negative control treated wells. Nuclei were detected using the “find nuclei” building block in the Hoechst channel, border objects were removed to ensure only whole nuclei were analysed. Micronuclei (MN) were detected using the “find Micronuclei” building block, and were filtered to align with the MN scoring criteria described by Fenech (2007)^63^. The “find spots” building block was utilised for kinetochore and for γ.H2AX foci analysis. Staining intensity and morphological properties were calculated for nuclei in all channels and pan γ.H2AX nuclei were determined from nuclear intensity properties.

The csv files of output data from Columbus were annotated with compound information and cell cycle profiles analysed using the R 3.6.0 software^64^. Cell-cycle distribution was estimated using Hoechst intensity measurements at the single nuclei level and an adaption the Dean-Jett-Fox algorithm^27^, which assumed all G1 cells had 2 N DNA, all G2 cells had 4 N DNA, with S phase cells distributed between. It was assumed all measurements are perturbed by normally distributed random noise. Cell-cycle distributions were fitted to DMSO control cells and calculated as follows: the G1 to G2 peak ratio was fixed to account for the inherent noise generated by immunofluorescence data, the standard deviation of the noise was assumed to be equal over the whole range of measurements; cells more than two standard deviations below the G1 peak were classified as Sub G1; cells within two standard deviations of the G1 peak and cells above two standard deviations from G1, but below 2 standard deviations from the G2 peak were classified as S; cells within 2 standard deviations of the G2 peak were classified as G2 and those greater than 2 standard deviations above the G2 peak were classified as greater than 4 N. All parameters were optimised simultaneously using the Nelder–Mead algorithm^65^.

This use of Hoechst channel intensities for cell-cycle analysis was validated by treatment of A549 cells for 24 h, 48 h, or 24 h (+ 24-h recovery period) with cell-cycle modulators using the same treatment and wash methods as above. Immunofluorescence was completed as above using Cyclin A (1:100, Abcam) and Cyclin D (1: 1000, Abcam) antibodies. DNA synthesis analysis was completed using the Click-iTTM EDU Alexa Fluro 488 Imaging Kit and the Click- iTTM reaction cocktail (ThermoFisher Scientific) followed by incubation with DNA stain FxCycle Violet (ThermoFisher Scientific) for 1 h.

### Well-masking procedure

Compound precipitation can interfere with micronucleus identification. To identify wells that contained precipitated compounds, the number of micronuclei/well was plotted against compound concentration. Excessive increases in micronuclei numbers, which could not be explained by genotoxic activity (for example, an increase from 50 to 15,000 micronuclei/well upon a two-fold increase of compound concentration), were flagged as potentially containing compound precipitation. The flagged wells were omitted from the analysis (CC50 exemplar selection, see below).

### Statistical workflow for genotoxicity prediction

The statistical framework for data processing and analysis was developed using Python 3 and consisted of three consecutive steps:

1. CC50 exemplar selection algorithm
  For CC50 exemplar selection, the mean cell number in the DMSO control wells was calculated per plate. This was utilised as the assumed “expected” cell number for each well on the plate, if the test compound had no effect on cell number. For each well, the ratio of the number of cells in that well (“observed”) compared to the expected number of cells was calculated. Wells or with an observed/expected ratio ≤ 0.5 were flagged as cytotoxic. In order to minimise potential experimental errors that may confound the results, for example pipetting or washing errors in a single well or position in a plate or imaging artefacts that may artificially alter cell number, a data smoothing algorithm scanned the assigned CC50 labels for each 15 point dose response. A cytotoxic concentration surrounded by two negative, non-cytotoxic concentrations, was exchanged for non-cytotoxic label as in such cases it was likely that such a response was due to well-to-well variability, rather than genuine cytotoxicity. If none of the fifteen concentrations were assigned cytotoxic label, the algorithm assigned CC50 to be the highest concentration.
  After selecting one CC50 exemplar for each replicate, a well-masking routine was employed. Briefly, for every well that was masked (due to compound precipitation), the algorithm removed that well and all wells with higher concentrations of that compound and set the highest remaining concentration as the CC50 exemplar.
2. Genotoxicity activity flagging
  To identify genotoxic compounds, a simple rule-based approach was employed. In line with the validated thresholds for genotoxic potential identification, an individual well was classified as “genotoxic” if at least a three-fold increase in the mean number of micronuclei/cells compared to the plate DMSO controls was observed. A fold increase between two and three-fold the number of micronuclei was flagged as a “borderline genotoxic” response. Compounds with less than a two-fold increase in the proportion of micronuclei containing cells were considered “non-genotoxic”.
3. Genotoxicity mechanism prediction
  An unsupervised machine learning method was employed to assign scores for genotoxic compound mechanisms of action; aneugenic, clastogenic or mixed mode of genotoxicity. Two features were employed for this task: the mean number of micronuclei with kinetochore per each cell (abbreviated further as F1, hallmark of aneugenicity) and the mean number of γ.H2AX foci per nucleus (F2, hallmark of clastogenicity). For each assay plate, two univariate Gaussian curves were fitted using Python 3 SciPy package^66^ to the F1 and F2 values of the intra-plate aneugen and clastogen controls, respectively. The estimated standard deviations of the Gaussians were then multiplied by a factor of 3. A compound with F1 value equal or higher than the mean of the aneugen control was automatically assigned a maximal score of 1.0 of being an aneugen. If the compound’s F1 value was below the mean of the aneugen control, the Gaussian probability density function (PDF) at that point was evaluated and the resulting likelihood was converted to a score by dividing it by the maximal likelihood of that PDF (a score of 0.0 meaning a non-aneugenic and 1.0 meaning a highly aneugenic compound). The same procedure was then repeated for the F2 values of each compound to calculate the clastogenicity score using the respective clastogen plate controls. Finally, the mechanism predictions of the two replicates were aggregated using mean.

Python code for this algorithm and an example data set is provided in supplementary data file 1.

## Supplementary Information

Supplementary Figures.

## Data Availability

Commercially available Python 3 SciPy package was utilised in this study.

## References

[1] Mattiazzi Usaj M (2016). High-content screening for quantitative cell biology. Trends Cell Biol..

[2] Murphy KP (2012). Machine Learning: A Probabilistic Perspective.

[3] Sieber OM, Heinimann K, Tomlinson IP (2003). Genomic instability—the engine of tumorigenesis?. Nat. Rev. Cancer.

[4] Guideline, I. H. T. in *International Conference on Harmonization of Technical Requirements for Registration of Pharmaceuticals for Human Use. ICH Expert Working Group.* 1–25.

[5] Diaz D, Scott A, Carmichael P, Shi W, Costales C (2007). Evaluation of an automated in vitro micronucleus assay in CHO-K1 cells. Mutat. Res..

[6] Mondal MS (2010). High-content micronucleus assay in genotoxicity profiling: Initial-stage development and some applications in the investigative/lead-finding studies in drug discovery. Toxicol. Sci..

[7] Sun B (2013). Assessing dose-dependent differences in DNA-damage, p53 response and genotoxicity for quercetin and curcumin. Toxicol. In Vitro Int. J. Publ. Assoc. BIBRA.

[8] Wilde EC (2018). A novel, integrated in vitro carcinogenicity test to identify genotoxic and non-genotoxic carcinogens using human lymphoblastoid cells. Arch. Toxicol..

[9] Bryce SM, Bernacki DT, Bemis JC, Dertinger SD (2016). Genotoxic mode of action predictions from a multiplexed flow cytometric assay and a machine learning approach. Environ. Mol. Mutagen..

[10] Hendriks G (2016). The extended ToxTracker assay discriminates between induction of DNA damage, oxidative stress, and protein misfolding. Toxicol. Sci..

[11] Kopp B, Khoury L, Audebert M (2019). Validation of the γH2AX biomarker for genotoxicity assessment: A review. Arch. Toxicol..

[12] Food & Drug Administration, H (2012). International conference on harmonisation; guidance on S2 (R1) genotoxicity testing and data interpretation for pharmaceuticals intended for human use; availability. Federal Reg..

[13] Roberts K (2016). Implementation and challenges of direct acoustic dosing into cell-based assays. J. Lab. Autom..

[14] Lorge E (2010). Comparison of different cytotoxicity measurements for the in vitro micronucleus assay using L5178Y and TK6 cells in support of OECD draft Test Guideline 487. Mutat. Res. Genet. Toxicol. Environ. Mutagen..

[15] Chambliss AB, Wu P-H, Chen W-C, Sun SX, Wirtz D (2013). Simultaneously defining cell phenotypes, cell cycle, and chromatin modifications at single-cell resolution. FASEB J..

[16] Chan GKY, Kleinheinz TL, Peterson D, Moffat JG (2013). A simple high-content cell cycle assay reveals frequent discrepancies between cell number and ATP and MTS proliferation assays. PLoS One.

[17] Tu W-Z (2013). γh2AX foci formation in the absence of DNA damage: Mitotic H2AX phosphorylation is mediated by the DNA-PKcs/CHK2 pathway. FEBS Lett..

[18] Lynch A, Parry J (1993). The cytochalasin-B micronucleus/kinetochore assay in vitro: Studies with 10 suspected aneugens. Mutat. Res. Fundam. Mol. Mech. Mutagen..

[19] Becker P, Scherthan H, Zankl H (1990). Use of a centromere-specific DNA probe (p82H) in nonisotopic in situ hybridization for classification of micronuclei. Genes Chromosom. Cancer.

[20] Kirkland D (2016). Updated recommended lists of genotoxic and non-genotoxic chemicals for assessment of the performance of new or improved genotoxicity tests. Mutat. Res. Genet. Toxicol. Environ. Mutagen..

[21] Kirkland D, Kasper P, Muller L, Corvi R, Speit G (2008). Recommended lists of genotoxic and non-genotoxic chemicals for assessment of the performance of new or improved genotoxicity tests: A follow-up to an ECVAM workshop. Mutat. Res..

[22] Ghoshal K, Jacob ST (1997). An alternative molecular mechanism of action of 5-fluorouracil, a potent anticancer drug. Biochem. Pharmacol..

[23] Nikolova T (2014). The γH2AX assay for genotoxic and nongenotoxic agents: Comparison of H2AX phosphorylation with cell death response. Toxicol. Sci..

[24] Moeglin E (2019). Uniform widespread nuclear phosphorylation of histone H2AX is an indicator of lethal DNA replication stress. Cancers.

[25] de Feraudy S, Revet I, Bezrookove V, Feeney L, Cleaver JE (2010). A minority of foci or pan-nuclear apoptotic staining of γH2AX in the S phase after UV damage contain DNA double-strand breaks. Proc. Natl. Acad. Sci..

[26] Brüsehafer K (2015). The clastogenicity of 4NQO is cell-type dependent and linked to cytotoxicity, length of exposure and p53 proficiency. Mutagenesis.

[27] Fox MH (1980). A model for the computer analysis of synchronous DNA distributions obtained by flow cytometry. Cytometry.

[28] Walko CM, Lindley C (2005). Capecitabine: A review. Clin. Ther..

[29] Yang K, Hitomi M, Stacey DW (2006). Variations in cyclin D1 levels through the cell cycle determine the proliferative fate of a cell. Cell Div..

[30] Pagano M, Pepperkok R, Verde F, Ansorge W, Draetta G (1992). Cyclin A is required at two points in the human cell cycle. EMBO J..

[31] Henglein B, Chenivesse X, Wang J, Eick D, Brechot C (1994). Structure and cell cycle-regulated transcription of the human cyclin A gene. Proc. Natl. Acad. Sci..

[32] Yam C, Fung T, Poon R (2002). Cyclin A in cell cycle control and cancer. Cell. Mol. Life Sci..

[33] Économiques ODCEDD (2016). Test No 487. In Vitro Mammalian Cell Micronucleus Test.

[34] Cheung JR (2015). Histone markers identify the mode of action for compounds positive in the TK6 micronucleus assay. Mutat. Res. Genet. Toxicol. Environ. Mutagen..

[35] Ayers KM, Clive D, Tucker JWE, Hajian G, de Miranda P (1996). Nonclinical toxicology studies with Zidovudine: Genetic toxicity tests and carcinogenicity bioassays in mice and rats. Fundam. Appl. Toxicol..

[36] Bolt HM, Degen GH (2004). Human carcinogenic risk evaluation, part II: Contributions of the EUROTOX specialty section for carcinogenesis. Toxicol. Sci..

[37] Elhajouji A, Lukamowicz M, Cammerer Z, Kirsch-Volders M (2011). Potential thresholds for genotoxic effects by micronucleus scoring. Mutagenesis.

[38] Wills JW, Johnson GE, Battaion HL, Slob W, White PA (2017). Comparing BMD-derived genotoxic potency estimations across variants of the transgenic rodent gene mutation assay. Environ. Mol. Mutagen..

[39] Joseph P (2009). Mechanisms of cadmium carcinogenesis. Toxicol. Appl. Pharmacol..

[40] Beyersmann D, Hechtenberg S (1997). Cadmium, gene regulation, and cellular signalling in mammalian cells. Toxicol. Appl. Pharmacol..

[41] Fowler P (2010). Cadmium chloride, benzo[a]pyrene and cyclophosphamide tested in the in vitro mammalian cell micronucleus test (MNvit) in the human lymphoblastoid cell line TK6 at Covance laboratories, Harrogate UK in support of OECD draft Test Guideline 487. Mutat. Res..

[42] Fellows MD, O'Donovan MR (2010). Etoposide, cadmium chloride, benzo[a]pyrene, cyclophosphamide and colchicine tested in the in vitro mammalian cell micronucleus test (MNvit) in the presence and absence of cytokinesis block using L5178Y mouse lymphoma cells and 2-aminoanthracene tested in MNvit in the absence of cytokinesis block using TK6 cells at AstraZeneca UK, in support of OECD draft Test Guideline 487. Mutat. Res..

[43] Hartwig A (1994). Role of DNA repair inhibition in lead- and cadmium-induced genotoxicity: A review. Environ. Health Perspect..

[44] Fotakis G, Cemeli E, Anderson D, Timbrell JA (2005). Cadmium chloride-induced DNA and lysosomal damage in a hepatoma cell line. Toxicol. In Vitro.

[45] Ravindran G, Chakrabarty D, Sarkar A (2017). Cell specific stress responses of cadmium-induced cytotoxicity. Anim. Cells Syst..

[46] Zapór L (2014). Evaluation of the toxic potency of selected cadmium compounds on A549 and CHO-9 cells. Int. J. Occup. Saf. Ergon..

[47] Longley DB, Harkin DP, Johnston PG (2003). 5-Fluorouracil: Mechanisms of action and clinical strategies. Nat. Rev. Cancer.

[48] Sirbu BM (2011). Analysis of protein dynamics at active, stalled, and collapsed replication forks. Genes Dev..

[49] Khoury L, Zalko D, Audebert M (2016). Evaluation of four human cell lines with distinct biotransformation properties for genotoxic screening. Mutagenesis.

[50] Bryce SM (2017). Interlaboratory evaluation of a multiplexed high information content in vitro genotoxicity assay. Environ. Mol. Mutagen..

[51] Callen E (2013). 53BP1 mediates productive and mutagenic DNA repair through distinct phosphoprotein interactions. Cell.

[52] Matsuzaki K, Harada A, Takeiri A, Tanaka K, Mishima M (2010). Whole cell-ELISA to measure the γH2AX response of six aneugens and eight DNA-damaging chemicals. Mutat. Res. Genet. Toxicol. Environ. Mutagen..

[53] Prigent C, Dimitrov S (2003). Phosphorylation of serine 10 in histone H3, what for?. J. Cell Sci..

[54] Bryce SM (2014). Interpreting in vitro micronucleus positive results: Simple biomarker matrix discriminates clastogens, aneugens, and misleading positive agents. Environ. Mol. Mutagen..

[55] Banerjee P, Siramshetty VB, Drwal MN, Preissner R (2016). Computational methods for prediction of in vitro effects of new chemical structures. J. Cheminform..

[56] Xu C (2012). In silico prediction of chemical Ames mutagenicity. J. Chem. Inf. Model..

[57] Cassano A (2014). Evaluation of QSAR models for the prediction of ames genotoxicity: A retrospective exercise on the chemical substances registered under the EU REACH regulation. J. Environ. Sci. Health Part C.

[58] Foster KA, Oster CG, Mayer MM, Avery ML, Audus KL (1998). Characterization of the A549 cell line as a type II pulmonary epithelial cell model for drug metabolism. Exp. Cell Res..

[59] Ooka M, Lynch C, Xia M (2020). Application of in vitro metabolism activation in high-throughput screening. Int. J. Mol. Sci..

[60] Olaharski AJ (2009). Identification of a kinase profile that predicts chromosome damage induced by small molecule kinase inhibitors. PLoS Comput. Biol..

[61] Johansson J, Larsson MH, Hornberg JJ (2019). Predictive in vitro toxicology screening to guide chemical design in drug discovery. Curr. Opin. Toxicol..

[62] Toledo L, Neelsen KJ, Lukas J (2017). Replication catastrophe: When a checkpoint fails because of exhaustion. Mol. Cell.

[63] Fenech M (2007). Cytokinesis-block micronucleus cytome assay. Nat. Protoc..

[64] Team, R. C. (2016).

[65] Nelder JA, Mead R (1965). A simplex method for function minimization. Comput. J..

[66] Virtanen P (2020). SciPy 1.0: Fundamental algorithms for scientific computing in Python. Nat. Methods.

