# Supplementary material for: Transforming early pharmaceutical assessment of genotoxicity: applying statistical learning to a high throughput, multi end point in vitro micronucleus assay
Source: Sci Rep. 2021 Jan 28;11:2535. doi: 10.1038/s41598-021-82115-5 (PMC7844000; doi:10.1038/s41598-021-82115-5)
Supplement: Supplementary file 1 — Supplementary Figures. [file 41598_2021_82115_MOESM1_ESM.docx]

**Transforming early pharmaceutical assessment of Genotoxicity: Applying statistical learning to a high throughput, multi end point in vitro micronucleus assay.**

Authors: Amy Wilson^1^, Piotr Grabowski^2^, Joanne Elloway^1^, Stephanie Ling^2^, Jonathan Stott^3^ and Ann Doherty^1^.

1. Functional and Mechanistic Safety, Clinical Pharmacology & Safety Sciences, R&D, AstraZeneca, Cambridge, United Kingdom.

2. Imaging and Data Analytics, Clinical Pharmacology & Safety Sciences, R&D, AstraZeneca, Cambridge, United Kingdom.

3. MAG-O, Manchester Airport, Manchester (current address)

Corresponding Author: amy.wilson3@astrazeneca.com

**SUPPLEMENTARY FIGURES**


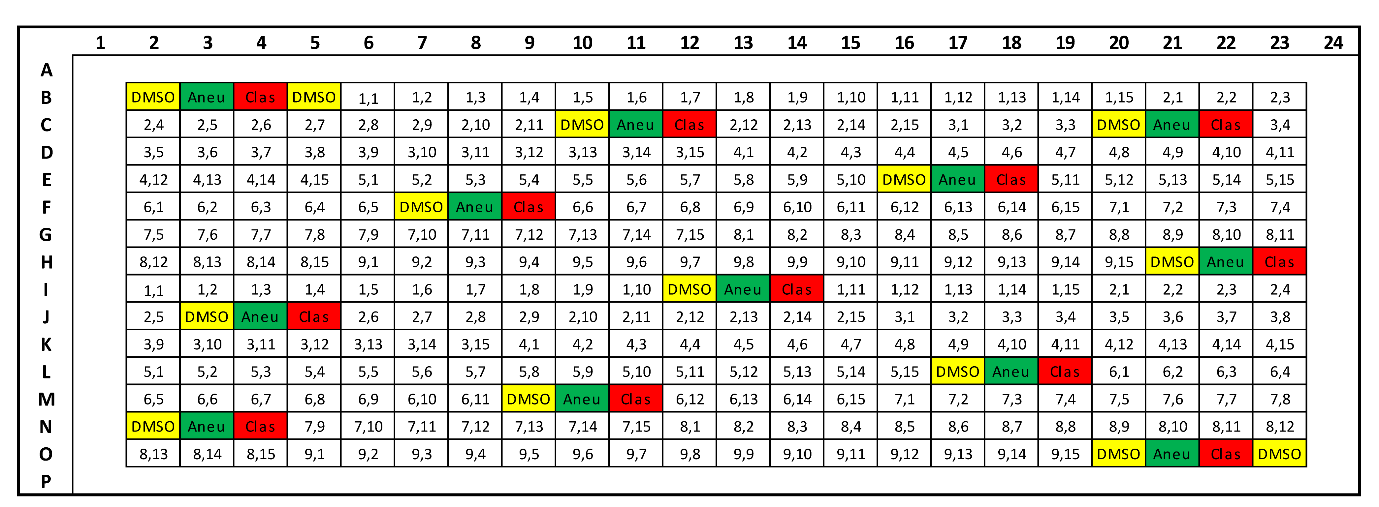


S.Fig 1: Plate map illustrating compound distribution in 384 well plate. Each numerical value represents (compound number (1-9), compound concentration(1-15)).Yellow wells indicate randomly distributed negative control (DMSO 2%) wells, Green, inter-plate Aneugenic control (Paclitaxel 2.5nM), and Red, Clastogenic controls (Etoposide 0.35µM).


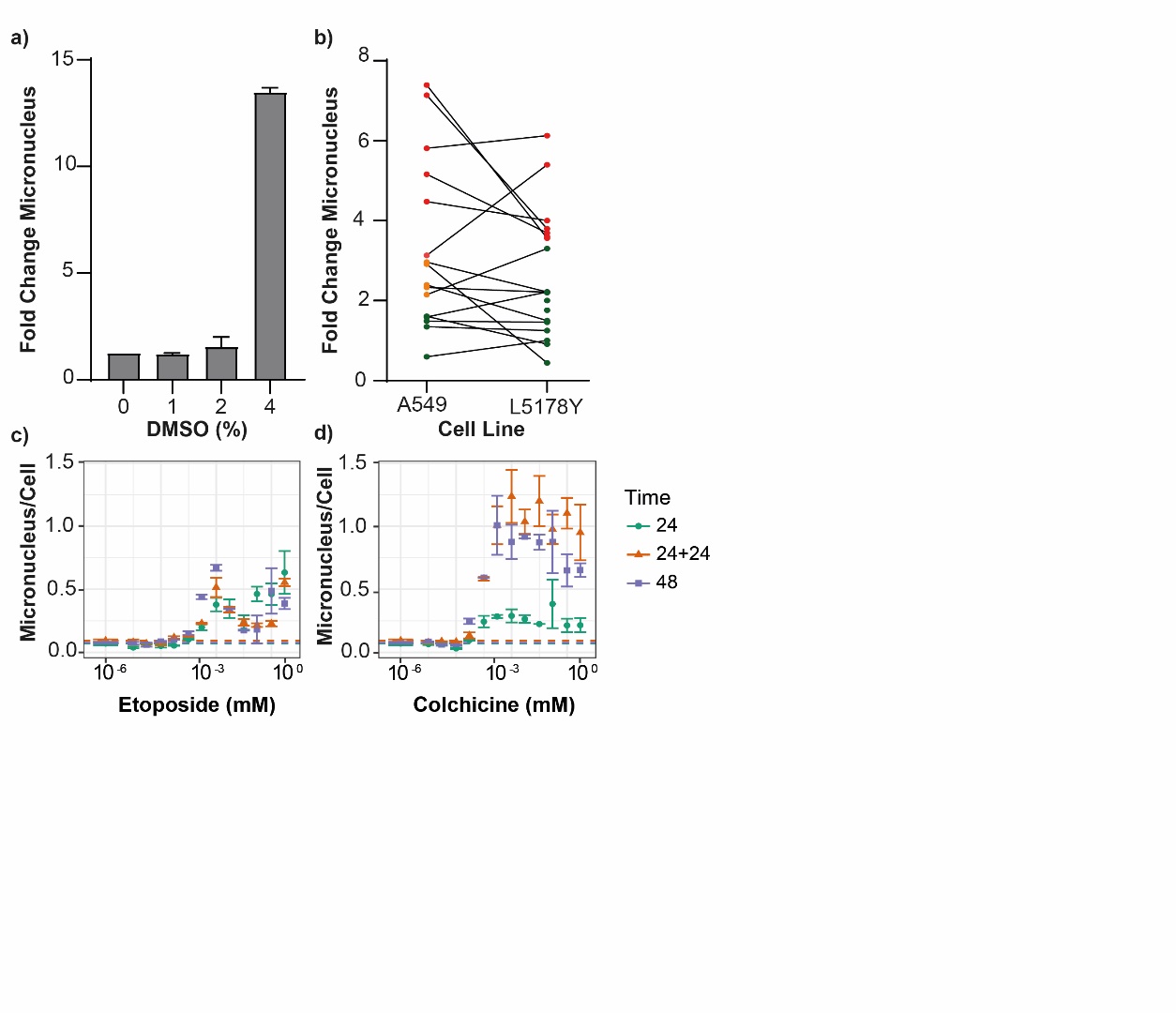


S.Fig 2: Rationale for increasing DMSO concentration. (a) Fold change in micronucleus frequency in A549 cells (compared to non-treated cells) after incubation for 24h with increasing % of DMSO. Comparison of A549 cell assay to regulatory L5178Y assay. (b) Fold change in micronucleus frequency (compared to DMSO control) of 16 AZ ‘compounds in A549 and L5178Y (mouse lymphoma) cell lines. Fold change in micronucleus frequency at IC50 dose (dose which induced approximately 50% cytotoxicity) is shown. Red dots indicate a positive response, orange a borderline response and green a negative response in the corresponding assays. Rationale for assay scheduling. (c) Representative responses of number of micronuclei normalised to cell number (expressed as micronuclei/cell) after treatment with a pro-typical Clastogen (Etoposide) and Aneugen (Colchicine) for 24h or 48h continuously and 24h followed by a 24h compound free recovery period.
